# 

**Published:** 1909-01-16

**Authors:** 


### GRADUATES' MEDICAL SCHOOLS.

DIARY FOR WEEK JAN. 18 TO JAN. 23.

### NATIONAL HOSPITAL FOR THE PARALYSED AND EPILEPTIC, Queen Sq., Bloomsbury, W.C.

**At 3.30 p.m.**
**Jan. 19,** Sir Wm. Gowers, F.R.S., **Myopathy and Syringomyelia.**
**Jan. 22,** Dr. Turner, **Epilepsy.**

### THE POST-GRADUATE COLLEGE, West London Hospital, Hammersmith, W.

**At 10 a.m.**
**Jan. 18 and 21,** Surgical Registrar, **Demonstration.**
**Jan. 22,** Medical Registrar, **Demonstration.**
**At 12 noon.**
**Jan. 18,** Dr. Low, **Pathological Demonstration.**
**At 12.15 p.m.**
**Jan. 20 and 23,** Dr. Pritchard, **Practical Medicine.**
**At 5 p.m.**
**Jan. 18,** Dr. Saunders, **Clinical Lecture.**
**Jan. 19,** Mr. Armour, **Clinical Lecture.**
**Jan. 20,** Mr. Baldwin, **Practical Surgery.**
**Jan. 21,** Dr. Davis, **Diseases of Throat, Nose, and Ear.**
**Jan. 22,** Dr. R. H. Cole, **General Paralysis.**

### THE THROAT HOSPITAL, Golden Square, W.

**At 5.30 p.m.**
**Jan. 18,** Mr. Faulder, **Anatomy and Physiology of Pharynx and Larynx.**
**Jan. 21,** Mr. Rose, **Methods of Examination, Local Applications.**

### ST. JOHN'S HOSPITAL FOR DISEASES OF THE SKIN, Leicester Square, W.C.

**Jan. 21,** Chesterfield Lectures, **Paratuberculides.**

### HOSPITAL FOR CONSUMPTION AND DISEASES OF THE CHEST, Brompton, S.W.

**Jan. 20,** Dr. Batty Shaw, **Occult Cardiac Hypertrophy.**

### THE INFANTS HOSPITAL, Vincent Square, Westminster.

**At 3.30 p.m.**
**Jan. 19, 21, and 22,** Dr. Vincent, **Demonstrations and Experimental Work in Research Laboratory.**

### NORTH-EAST LONDON POST-GRADUATE COLLEGE, Prince of Wales's General Hospital, Tottenham, N.

**At 4.30 p.m.**
**Jan. 19,** Dr. A. J. Whiting, **Aortic Valvular Disease.**
**Jan. 21,** Mr. H. W. Carson, **The After-results of Gastro-Enterostomy.**

### UNIVERSITY COLLEGE HOSPITAL.

An esteemed correspondent, writing with authority, sends us the following communication. We have dealt in another column of the present issue with the important bearings of this appointment :—

No more appropriate appointment could have been made to the important post of Secretary to University College Hospital than that of Mr. J. G. Buckle, the Assistant Secretary of the *Dreadnought Hospital* at Greenwich. Mr. Buckle is a man of great practical experience. He went with a scholarship from Malvern to Magdalen, Cambridge, and then passed third into the Colonial Service, and was stationed for a number of years at Hong Kong, holding the post of Assistant Colonial Secretary and Secretary to the Executive and Legislative Council. On his retirement from Government service he took office with the Chancellor of London University, and from that he became Assistant Secretary at King's College. He then entered the Seamen's Hospital at Greenwich. As Assistant Secretary in that Institution he has gained a thorough insight into the modern system of hospital administration, and University College may be congratulated on the choice they have made.

### ANSWERS TO CORRESPONDENTS.

Dr. J. C. Uhthoff is thanked for his letter, and the suggestion contained in it. The latter will, if possible, be acted upon at an early date.

